# Observation of the Gut Microbiota Profile in BALB/c Mice Induced by *Plasmodium yoelii* 17XL Infection

**DOI:** 10.3389/fmicb.2022.858897

**Published:** 2022-03-31

**Authors:** Wei Guan, Xiaonan Song, Shuguo Yang, Huiyin Zhu, Fang Li, Jian Li

**Affiliations:** ^1^Department of Human Parasitology, School of Basic Medicine Science, Hubei University of Medicine, Shiyan, China; ^2^Department of Infectious Diseases, Renmin Hospital, Hubei University of Medicine, Shiyan, China

**Keywords:** *Plasmodium yoelii*, malaria, gut microbiota, 16S rRNA, biomarker

## Abstract

Rodent malaria caused by *Plasmodium yoelii* 17XL (Py 17XL) is an ideal animal model for human malaria studies. Although the gut microbiota plays an important role in the occurrence and development of infectious diseases, the gut microbiota associated with Py 17XL infection remains unclear. In the current study, the gut microbiota composition of infected BALB/c mice was surveyed. Mouse fecal samples were collected at 0, 2, 5 days post-infection (dpi), and the gut microbiota was characterized by 16S rRNA sequencing. Operational taxonomic units (OTUs) were 634 ± 26 on average. *Firmicutes* and *Bacteroidetes* were typically predominant in the gut microbiota composition at the phylum level. Compared with the Ctrl, *Firmicutes* was significantly decreased after infection, while *Bacteroidetes* was notably increased. The most dominant family was *Lactobacillaceae* in all samples. The alpha diversity index showed that compared with that of the Ctrl, the observed OTU number was decreased at 2 dpi and then slightly increased at 5 dpi. LEfSe analysis revealed several bacterial taxa were notably related to Py-infected mice at the phylogenetic level. Several bacterial genera, such as *Lactobacillus*, were overrepresented in the Py*-*infected fecal microbiota at 2 dpi, while *Muribaculaceae* was overrepresented at 5 dpi. Moreover, *Alistipes and Helicobacter* were overrepresented at 5 dpi compared with 2 dpi. The results indicated Py infection could alter the gut microbiota composition of mice. Besides, biomarkers could serve as direct targets to elucidate their roles in the progression and pathogenesis of malaria and provide insights into studies of antimalarial drugs and malaria vaccines.

## Introduction

Malaria is an acute infectious disease that threatens the health of approximately half of the world’s population and was responsible for an estimated 241 million clinical cases and approximately 627,000 deaths in 2020 (WHO, 2021). Additionally, there were 14 million more cases in 2020 than in 2019 and 69,000 more deaths. Approximately two-thirds of these additional deaths (47,000) were linked to disruptions in the provision of malaria prevention, diagnosis and treatment during the COVID-19 pandemic (WHO, 2021). Malaria is caused by protozoan parasites of the genus *Plasmodium* and is the most prevalent infectious disease in the WHO African Region (De et al., 2016). China, on the other hand, was officially certified as a malaria-free country by the WHO in 2021 (Feng et al., 2021). Malaria confers a large economic burden and disease burden (Waide and Schmidt, 2020). Five main species of the genus *Plasmodium* can cause human malaria. Fever, chills, headache, muscle aches, and tiredness are the main symptoms of malaria patients. In addition, gastrointestinal symptoms such as vomiting, abdominal pain, and diarrhea develop in *falciparum* malaria patients (Prasad and Virk, 1993). Under laboratory or clinical conditions, different strains or lines of malaria parasites can cause different severities of malaria in their hosts. The rodent malaria parasite *Plasmodium yoelii* is a representative example of parasite lines with significantly different pathogenicity. BALB/c mice infected with the parasite Py 17XL die within 7 dpi (Nair et al., 2017). Rodent malaria caused by Py 17XL is a typical rodent model for studying the pathogenesis of human malaria (Chen et al., 2010). While there is still much to explore before gut microbiota modulation becomes an effective and optimal treatment for preventing fatal malaria, recent evidence in both human studies and rodent models has indicated the gut microbiota composition is a factor in the progression of disease.

Trillions of microbes naturally reside in the human body, especially in the gastrointestinal tract (Gill et al., 2006). The gut microbiota has many important functions in the human body, including resisting pathogens to support protection, enhancing the immune system, and contributing to digestion and metabolism (Gomaa, 2020). The disturbance of the gut microbiota community is associated with several human diseases, such as inflammatory bowel diseases (IBDs) (Nishino et al., 2018), obesity and diabetes (Karlsson et al., 2013), allergies (Bunyavanich et al., 2016), autoimmune diseases (Chu et al., 2017), and cardiovascular diseases (Astudillo and Mayrovitz, 2021). Moreover, there are a lot of methods for modulating the gut microbiota composition and functions, such as the application of probiotics and fecal microbiota transplantation. Previous studies have suggested the gut microbiota could also modulate the pathogenesis of infectious diseases, indicating that gut microbiota variability influences systemic immune responses. For instance, mice colonized with a gut pathobiont could produce antibodies that reacted with *Plasmodium spp.* (Yilmaz et al., 2014). Besides, gastrointestinal environments, especially changes in the gut microbiota, are related to the onset of several diseases.

Malaria infections affect the gastrointestinal tract, and alterations in the intestinal environment appear to influence malaria pathogenesis. Villarino et al. (2016) showed when different vendors’ C57BL/6 mice were infected with the non-lethal rodent-specific strain Py 17XNL, the mice presented numerous differences in infection morbidity, severity, and mortality and that these differences in susceptibility were dependent on the gut microbiota. Taniguchi et al. demonstrated that C57BL/6 mice infected with the lethal rodent-specific strain *P. berghei* ANKA developed experimental cerebral malaria. The alterations included intestinal pathology and gut microbiota composition, which were related to the development of ECM. Moreover, Denny et al. verified that proinflammatory cells were increased in the lamina propria and changes in cecal metabolites were observed with differing susceptibility to Py 17XNL (Denny et al., 2019). However, some *Plasmodium* parasites appear to have developed resistance to available antimalarials. Besides, there is no practical or long-term vaccine against malaria. The relationship between human health and disease and gut microbiota has been studied widely in recent years. Hence, gut microbiota modulation may be a potential treatment for malaria (Villarino et al., 2016).

Recent studies have reported that many various diseases can alter the gut microbiota, which led us to hypothesize that the gut microbiota may be affected by malaria infections. BALB/c mice with Py 17XL infection are a lethal malaria model that can be used to clarify the possible role of Py in influencing the gut microbiota of infected BALB/c mice. Therefore, the aims of the study included (1) analyzing the gut microbiota composition obtained from the feces of Py 17XL-infected and -uninfected mice separately by targeting the V4 region of the 16S rRNA through the Illumina HiSeq2500 platform and (2) obtaining biomarkers to offer insight into malaria pathogenesis and antimalarial drugs.

## Materials and Methods

### Mice and Infection With *Plasmodium yoelii* 17XL

Six- and eight-week-old female BALB/c mice (20-25 g weight) were purchased from HNSJA Co., Ltd., Changsha, China, and maintained under specific pathogen-free conditions. The housing and feeding conditions were maintained as per recommended standards (25 ± 3°C). The mice were fed an irradiated diet and pure water. All the mice were allowed to acclimate for one week before starting the experiment.

Donor female BALB/c mice were intraperitoneally infected with Py 17XL by infected red blood cells (iRBCs) prepared from thawed blood. Fresh blood was collected from donor mice at 4 dpi, and the experimental BALB/c mice were intraperitoneally infected with 1 × 10^6^ iRBCs (in 100 μl saline) prepared from the donor mice. Tail blood was collected for thin smears and stained with Giemsa dye at 2 and 5 dpi, respectively. Percent parasitemia assessed as percent iRBCs per total RBCs was evaluated through Giemsa-stained thin blood smears, and parasites versus whole blood cells were counted at 1,000 × magnification under a light microscope.

### Sample Collection

The sixteen experimental mice were placed in the cage individually. Fecal pellets were collected at three time points as follows: before infection with Py 17XL (Ctrl), at 2 days post-infection (PyD2), and at 5 days post-infection (PyD5). Mice feces were placed in a sterile centrifuge tube using a sterile tweezer, immediately frozen in liquid nitrogen and stored at −80°C until use.

#### DNA Extraction, PCR Amplification, and Sequencing

DNA was extracted from the fecal samples using the CTAB or SDS method. PCR amplification and sequencing were performed using the microbial 16S rRNA primers (16S V4: 515F-806R, 392 bp) as well as Phusion^®^ High-Fidelity PCR Master Mix (New England Biolabs). The PCR product mixture was purified using a Qiagen Gel Extraction Kit (Qiagen, Germany).

Sequencing libraries were generated using a TruSeq^®^ DNA PCR-Free Sample Preparation Kit (Illumina, United States). The library quality was evaluated on the Qubit@ 2.0 Fluorometer (Thermo Scientific) and Agilent Bioanalyzer 2100 system. Finally, the library was sequenced on an Illumina HiSeq2500 platform. The 16S rRNA amplicons sequenced in this study were uploaded into the Sequence Read Archive (SRA) in NCBI and registered with the BioProject database (BioProject identification number PRJNA798515).

### Data Processing and Statistical Analysis

Data processing and statistical analysis methods followed (Guan et al., 2021). Paired-end reads were merged using FLASH V1.2.7 (Magoč and Salzberg, 2011). Sequence analysis was performed using QIIME (V1.7.0) (Caporaso et al., 2010). The chimera sequences were determined using the UCHIME algorithm (Edgar et al., 2011) and were removed (Haas et al., 2011) to obtain the effective tags. The operational taxonomic units (OTUs) were determined by using Uparse software (Uparse v7.0.1001) (Edgar, 2013) and assigned by sequences with ≥ 97% similarity. Representative sequences for each OTU were screened for further annotation. For each representative sequence, the GreenGene Database (DeSantis et al., 2006) was used based on the RDP 3 classifier (Version 2.2) (Wang et al., 2007) algorithm to annotate the taxonomic information. To study the phylogenetic relationships of the different OTUs and the differences among the dominant species in different groups, multiple sequence alignment was conducted using MUSCLE software (Version 3.8.31) (Edgar, 2004).

Operational taxonomic units abundance information was normalized using a standard sequence number corresponding to the sample with the fewest sequences. Statistical analysis was performed by alpha diversity and beta diversity. The alpha diversity was applied to analyze the complexity of the species diversity for a sample based on six indices: Chao1, ACE, Observed-species, Shannon, Simpson, and Goods-coverage. All of these indices in our samples were calculated with QIIME (Version 1.7.0) and displayed with R software (Version 2.15.3).

In contrast, the beta diversity was evaluated by determining the similarity among the microbial communities. Both weighted and unweighted UniFrac calculations were performed by using QIIME software (Version 1.7.0). Principal coordinate analysis (PCoA) was performed to obtain the principal coordinates and visualize the complex, multidimensional data. The WGCNA package, stat packages, and ggplot2 package in R software (Version 2.15.3) were applied for PCoA analysis. The unweighted pair-group method with arithmetic means (UPGMA) clustering and LEfSe were performed to obtain differentially represented microbial taxa at different taxonomic levels.

## Results

### General Information

**FIGURE 1:**
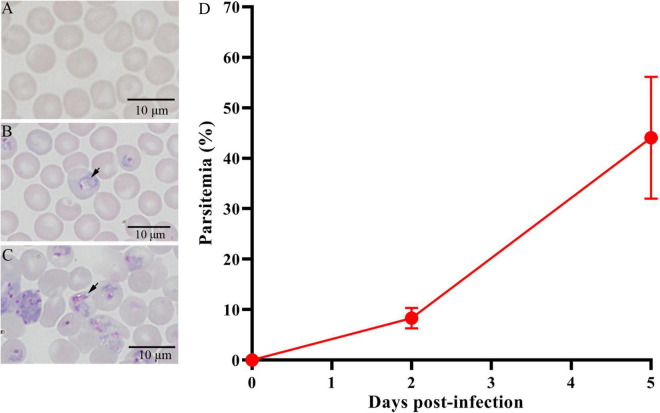
Giemsa’s stain to determine the parasitemia of Py 17XL in BALB/c mice. **(A)** The control mice (Ctrl) without parasite infection. **(B)** The PyD2 group with successful parasite infection at 2 dpi. **(C)** The PyD5 group with successful parasite infection at 5 dpi. **(D)** Percent parasitemia was evaluated on the corresponding days. Data are cumulative results (*n* = 16 mice per group) from two experiments.

On day 0, normal mice (Figure 1A) were infected with Py 17XL parasites. At 2 and 5 dpi, the parasites were observed in thin blood smears using microscopy, representing for successful infection (Figures 1B,C). The parasitemia of the infected mice at 5 dpi was higher than that of the infected mice at 2 dpi (Figure 1D). The feces were collected on day 0, day 2, and day 5 after infection.

### Sequencing and Operational Taxonomic Units Clustering

A total of 48 mouse fecal samples were collected and sent for 16S RNA sequencing. In total, 3,805,568 raw sequences were generated from all the samples, and the sequence number varied from 70,216 to 88,910. The mean number of sequences was 79,283 ± 5,941 (standard deviation, SD) per sample. After a series of experiments, a total of 3,428,267 clean sequences remained, which ranged from 62,444 to 82,917 sequences per sample, with an average of 71,422 ± 5,514 (Table 1). The GC content in all the samples ranged from 51.78% to 53.73%, and the average was 52.68%. The high-quality reads were clustered using ≥ 97% sequence similarity into a total of 30,428 microbiota OTUs, ranging from 538 to 686. Each sample had 634 OTUs, 594 observed species, and 71,422 sequences on average (Table 1).

**TABLE 1:** Operational taxonomic unit (OTU)-based diversity indexes in mice gut samples during infection.

Sample Name	Group	Raw PE(#)	Nochime (#)	Base(nt)	GC%	Effective%	OTU_ num	Observed_ species	Shannon	Simpson	Chao1	ACE	Goods_ coverage
PyD0.1	Ctrl	70,337	64,174	16,214,197	52.19	91.24	644	606	6.34	0.95	624.32	628.43	0.999
PyD0.2		84,273	73,165	18,487,519	53.14	86.82	674	632	6.87	0.97	658.52	662.18	0.999
PyD0.3		75,412	67,408	17,038,027	52.48	89.39	646	597	6.04	0.94	642.22	632.98	0.999
PyD0.4		84,840	73,283	18,522,540	52.84	86.38	658	613	6.20	0.95	634.36	643.35	0.999
PyD0.5		75,051	65,766	16,619,156	52.82	87.63	614	575	5.37	0.88	616.64	619.24	0.999
PyD0.6		87,986	75,056	18,971,697	53.61	85.3	634	600	6.01	0.93	626.02	625.12	0.999
PyD0.7		83,609	76,237	19,263,197	52.39	91.18	656	611	6.73	0.98	631.57	640.12	0.999
PyD0.8		86,624	79,942	20,204,281	52.46	92.29	643	592	4.75	0.79	614.61	634.14	0.999
PyD0.9		87,613	77,875	19,672,839	53.25	88.89	661	620	6.14	0.93	665.12	662.65	0.999
PyD0.10		75,867	65,990	16,678,473	52.57	86.98	647	601	4.74	0.76	635.88	637.08	0.999
PyD0.11		87,356	78,520	19,832,348	53.07	89.89	674	629	6.99	0.98	648.80	651.66	0.999
PyD0.12		86,098	78,866	19,923,106	52.78	91.6	656	618	6.51	0.96	638.61	652.30	0.999
PyD0.13		76,600	70,510	17,807,037	52.64	92.05	634	589	6.67	0.98	631.14	622.20	0.999
PyD0.14		70,749	64,814	16,363,835	53.43	91.61	636	603	6.68	0.98	631.52	636.62	0.999
PyD0.15		71,469	62,444	15,775,047	53.06	87.37	616	584	6.42	0.96	595.23	602.89	0.999
PyD0.16		78,065	69,093	17,453,274	52.93	88.51	649	602	6.43	0.95	618.72	622.33	0.999
PyD2.1	PyD2	76,928	71,536	18,068,885	52.35	92.99	630	588	6.43	0.97	617.12	615.48	0.999
PyD2.2		71,918	63,493	16,042,265	52.44	88.29	632	599	5.72	0.93	635.40	628.44	0.999
PyD2.3		88,910	81,343	20,519,433	53.64	91.49	627	596	6.38	0.97	618.69	618.30	0.999
PyD2.4		85,378	77,337	19,536,102	52.66	90.58	665	629	5.68	0.91	657.60	667.61	0.999
PyD2.5		84,925	74,679	18,872,116	52.45	87.94	626	593	4.99	0.85	620.02	621.89	0.999
PyD2.6		74,062	70,345	17,767,323	52.45	94.98	578	541	5.71	0.94	569.50	572.46	0.999
PyD2.7		88,829	78,176	19,745,231	52.1	88.01	650	612	6.04	0.94	630.42	639.04	0.999
PyD2.8		79,308	71,996	18,183,311	52.58	90.78	617	577	5.31	0.90	628.94	617.79	0.999
PyD2.9		74,001	64,752	16,364,551	52.35	87.5	612	566	4.62	0.82	596.45	590.92	0.999
PyD2.10		74,817	63,958	16,157,138	52.13	85.49	614	570	5.31	0.91	615.18	609.11	0.999
PyD2.11		80,044	66,995	16,931,900	52.28	83.7	599	550	4.35	0.77	596.47	592.48	0.999
PyD2.12		80,153	70,532	17,827,502	51.84	88	624	580	5.64	0.94	601.28	603.31	0.999
PyD2.13		85,308	76,723	19,379,710	52.46	89.94	678	634	5.78	0.95	668.23	682.08	0.999
PyD2.14		77,261	68,905	17,411,193	52.09	89.18	624	588	5.48	0.91	620.50	627.89	0.999
PyD2.15		72,386	66,325	16,767,584	52.37	91.63	538	501	4.03	0.77	516.11	520.43	0.999
PyD2.16		79,307	72,830	18,401,804	52.58	91.83	622	578	4.73	0.79	608.61	608.17	0.999
PyD5.1	PyD5	79,934	73,460	18,558,327	52.54	91.9	623	586	6.84	0.98	612.73	610.75	0.999
PyD5.2		71,038	65,481	16,539,019	52.47	92.18	637	604	6.95	0.98	630.63	630.28	0.999
PyD5.3		77,717	72,077	18,207,313	52.96	92.74	619	588	6.49	0.97	613.11	615.72	0.999
PyD5.4		88,908	82,917	20,938,956	52.44	93.26	638	601	6.60	0.98	637.85	639.19	0.999
PyD5.5		71,624	66,342	16,759,865	52.75	92.63	624	594	6.52	0.96	614.57	623.67	0.999
PyD5.6		83,105	73,604	18,584,672	53.27	88.57	618	581	5.81	0.93	631.31	621.99	0.999
PyD5.7		84,200	79,036	19,958,270	52.39	93.87	611	569	6.23	0.97	591.64	601.91	0.999
PyD5.8		79,922	73,812	18,642,216	52.71	92.36	647	611	6.64	0.98	645.04	643.89	0.999
PyD5.9		77,161	70,971	17,926,955	52.07	91.98	630	597	6.47	0.97	617.63	620.75	0.999
PyD5.10		77,214	68,398	17,276,058	53.3	88.58	639	608	6.28	0.94	634.56	633.44	0.999
PyD5.11		70,216	63,661	16,078,482	52.69	90.66	618	593	6.60	0.97	616.02	616.95	0.999
PyD5.12		73,293	67,990	17,178,324	51.78	92.76	686	639	6.59	0.97	674.02	676.67	0.999
PyD5.13		84,219	77,320	19,527,987	53.1	91.81	616	569	6.43	0.96	595.91	606.35	0.999
PyD5.14		70,954	64,678	16,331,402	53.73	91.15	659	611	6.81	0.97	623.24	632.25	0.999
PyD5.15		85,073	77,047	19,469,881	52.87	90.57	635	597	6.03	0.93	614.75	625.17	0.999
PyD5.16		75,506	68,405	17,293,772	52.97	90.6	650	610	5.98	0.93	639.50	642.31	0.999
Total		3,805,568	3,428,267	866,074,120			30,428	28,532					
Maximum value		88,910	82,917	20,938,956	53.73	94.98	686	639	6.99	0.98	674	682	0.999
Minimum value		70,216	62,444	15,775,047	51.78	83.70	538	501	4.03	0.76	516	520	0.999
Average		79,283	71,422	18,043,211	52.68	90.11	634	594	6.01	0.93	623	626	0.999
SD		5941	5514	1391042	0.45	2.48	26	25	0.74	0.06	26	27	4E-16

*Ctrl, PyD2and PyD5 represent group control and Py 17XL-infected mice at 2 dpi, Py 17XL-infected mice at 5 dpi, respectively.*

**FIGURE 2:**
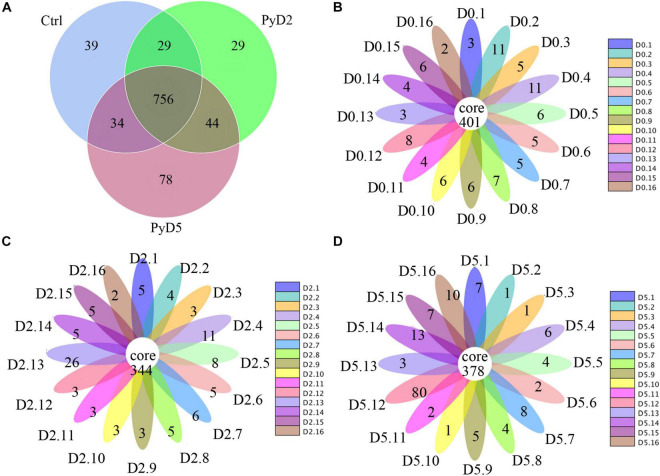
Operational taxonomic unit (OTU) analysis in the different fecal samples. **(A)** Venn diagram showing the unique and shared OTUs in the various groups and communities. **(B)** Flower diagram showing the shared and unique OTUs in the Ctrl samples. **(C)** Flower diagram showing the exclusive and mutual OTUs in the PyD2 samples. **(D)** The flower diagram shows the exclusive and mutual OTUs in the PyD5 samples.

In sum, the OTU number was decreased after Py infection compared with that in the control. Table 1 lists the detailed characteristics of each sample. The Venn diagram and flower diagram were generated to compare the similarities and differences between the communities in the different groups and samples. There were a total of 1,009 OTUs in all three groups, which had 756 common OTUs. There were 39, 29, and 78 OTUs unique to the Ctrl, PyD2, and PyD5 groups, respectively. The PyD2 group had equal OTUs to the Ctrl group. The PyD5 group had 54 more OTUs than the Ctrl group. Meanwhile, the PyD5 group had 54 more OTUs than the PyD2 group (Figure 2A). For individual samples, the maximum OTUs were 80 in PyD5.12, the minimum OTUs were 1 in PyD5.2, PyD5.3, and PyD5.10, and the mean number of unique OTUs was 7 (Figures 2B–D).

The species accumulation curves represented species richness in all samples that were close to the plateau phase (Supplementary Figure 1A). Likewise, the rarefaction curves indicated that species representation in individual samples was close to the saturation number of observed species (Supplementary Figure 1B).

#### Species Annotation and Taxonomic Overview

**FIGURE 3:**
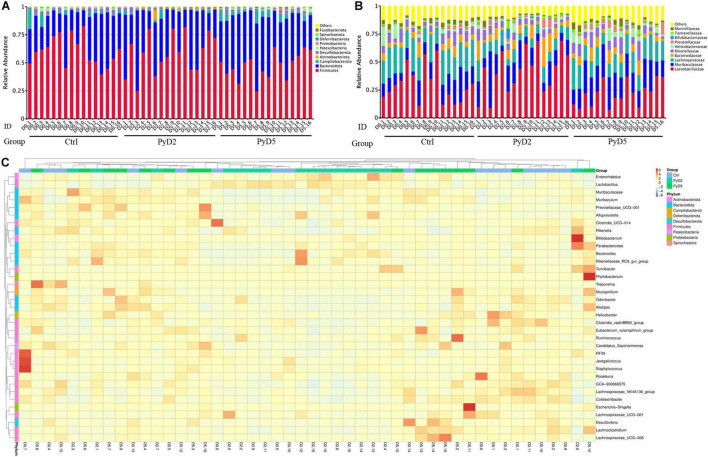
Changes in the gut microbiota composition. Relative abundance of fecal bacterial phyla and families existing in the BALB/c mice. Each bar represents the top 10 abundant phyla and families, whereas “Other” were obtained from low abundant and unclassified OTUs. Sample names represent samples described in Table 1. **(A)** Variations in the gut microbiota composition at the phylum level. **(B)** Variations in the gut microbiota composition at the family level; **(C)** Taxonomic heatmap of the three groups at the genus level. Typical relative **g10** abundance of diversified genera present in the Ctrl, PyD2, and PyD5 groups. Each column represents a unique subject.

Figure 3 shows the microbiota community composition of feces in all three groups of BALB/c mice. Each bar represents the average relative abundance of each microbial taxon in the stacked bar chart. The top 10 taxa with high relative abundance, which comprised 97% of the reads, are illustrated.

At the phylum level (Figure 3A), *Firmicutes* (55.39%) and *Bacteroidetes* (37.65%) were the major gut microbiota composition in all the samples. Compared with the Ctrl, *Firmicutes* was significantly decreased in post-infection samples, while *Bacteroidetes* was notably increased in post-infection samples.

At the family level (Figure 3B), the most dominant family was *Lactobacillaceae* (31.13%) in all the samples. *Muribaculaceae* (17.55%), *Lachnospiraceae* (16.53%), *Bacteroidaceae* (7.20%), *Rikenellaceae* (6.75%), *Helicobacteraceae* (3.11%), *Marinifilacea*e (2.53%), *Prevotellaceae* (2.19%), *Tannerellaceae* (1.43%), and *Bifidobacteriaceae* (0.30%) were the subdominant families. The abundance of *Muribaculaceae*, *Prevotellaceae*, and *Tannerellaceae* was increased in the post-infection samples compared with the Ctrl samples. The abundance of *Lactobacillaceae*, *Bacteroidaceae*, and *Bifidobacteriaceae* was dramatically increased at 2 dpi and then slightly decreased at 5 dpi. At the same time, the abundance of *Lachnospiraceae*, *Rikenellaceae*, *Helicobacteraceae*, and *Marinifilaceae* was slightly decreased at 2 dpi and then slightly increased at 5 dpi.

At the genus level, several taxa displayed significant differences among the three groups. Hierarchical clustering based on the abundance profile of the genera showed the most common genus in each group (Figure 3C). The gut microbiota in the samples of infected mice was characterized by higher abundances of *Lactobacillus*, *Muribaculaceae*, *Bacteroides*, *Alistipes*, *Lachnospiraceae_NK4A136_group*, *Helicobacter*, *Odoribacter*, *Alloprevotella*, *Parabacteroides*, and *Bifidobacterium*.

#### Analysis of the Bacterial Community Within Groups

Alpha diversity analysis was performed to explore the microbiota community diversity in the samples (within-group), reflecting the richness and diversity of the microbiota community. Alpha diversity metrics, including Chao1, Goods-coverage, observed species, Shannon and Simpson diversity index, and phylogenetic diversity values (Table 1), revealed a trend that infection with Py transiently changed the richness and evenness among the Ctrl, PyD2 and PyD5 groups. The estimated and observed OTUs in the three groups were different. The OTU number, observed species, and Shannon and Simpson index values were decreased at 2 dpi and then slightly increased at 5 dpi. Outliers were detected in all three groups (Supplementary Figure 2).

For Chao1 analysis (Supplementary Figure 2A), which represents an index for estimating the number of OTUs in the sample, the Ctrl samples had more estimated OTUs than the PyD2 group (*p* = 0.0833, *t*-test; *p* = 0.0289, Wilcox rank-sum test) and PyD5 group (*p* = 0.6761, *t*-test; *p* = 0.1617, Wilcox rank-sum test), while the PyD2 group had fewer OTUs than PyD5 group (*p* = 0.377, *t*-test; *p* = 0.408, Wilcox rank-sum test).

For observed species analysis (Supplementary Figure 2B), which represents the number of species contained in the sample, the Ctrl group samples had higher numbers than the PyD2 group (*p* = 0.0198, *t*-test; *p* = 0.0069, Wilcox rank-sum test) and the PyD5 group (*p* = 0.6650, *t*-test; *p* = 0.2384, Wilcox rank-sum test), and the PyD2 group had fewer observed species than the PyD5 group (*p* = 0.1388, *t*-test; *p* = 0.1084, Wilcox rank-sum test).

For Shannon diversity analysis (Supplementary Figure 2C), which assesses the richness and evenness of species composition in the sample, the samples of the Ctrl group had a higher number than the PyD2 group (*p* = 0.0012, *t*-test; *p* = 2.00E-04, Wilcox rank-sum test) but a lower number than the PyD5 group (*p* = 0.3987, *t*-test; *p* = 0.2132, Wilcox rank-sum test), while the PyD2 group had a lower number than the PyD5 group (*p* = 1.88E-05, *t*-test; *p* < 0.001, Wilcox rank-sum test).

For phylogenetic diversity analysis (Supplementary Figure 2D), which assesses the relationship of species in the microbiota community, samples in the Ctrl group had a slightly higher number than those in the PyD2 (*p* = 0.547, *t*-test) and PyD5 groups (*p* = 0.959, *t*-test), while the PyD2 group had a few lower numbers than the PyD5 group (*p* = 0.7182, *t*-test).

#### Analysis of the Bacterial Community Between Groups

For the unweighted UniFrac analysis (Supplementary Figure 2E), referring to the presence or absence of alterations in the species, beta diversity analysis was different among the Ctrl, PyD2, and PyD5 groups. The values in the PyD2 samples were increased relative to those in the Ctrl and PyD5 samples, but the values in the PyD5 samples were decreased relative to those in the Ctrl samples. That is, the samples in the PyD2 group had a higher number of OTUs than the Ctrl samples (*p* = 0.7244, *t*-test; *p* = 0.6495, Wilcoxon rank-sum test) and PyD5 samples (*p* = 0.7232, *t*-test; *p* = 0.0117, Wilcoxon rank-sum test). Moreover, the samples in the PyD5 group had a lower number of OTUs than the Ctrl group (*p* = 0.2843, *t*-test; *p* = 0.0382, Wilcoxon rank-sum test). It also demonstrated that the discreteness of the beta diversity increased from Ctrl to PyD2 and then decreased from PyD2 to PyD5. Meanwhile, for the weighted UniFrac analysis concerning both the presence or absence of species and the alterations in species abundance, the values among the Ctrl, PyD2, and PyD5 groups had an obviously similar trend to those of the unweighted UniFrac analysis (Supplementary Figure 2F).

**FIGURE 4:**
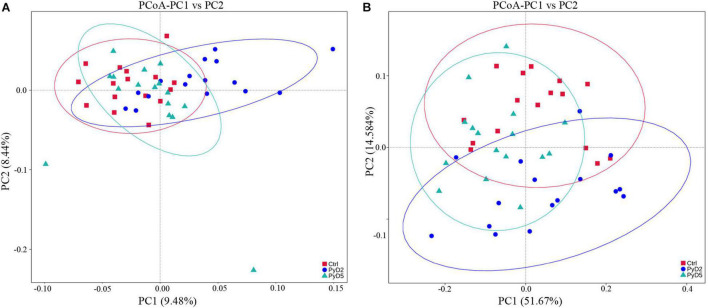
Principal coordinates analysis (PCoA) based on unweighted **(A)** and weighted **(B)** Unifrac distance matrices of fecal samples of BALB/c mice during infection. The fecal bacterial community components of both Py 17XL-infected and -uninfected mice were represented. Sample names are described in Table 1.

The similarity degree between the BALB/c mouse gut microbiota composition in each sample was examined using the principal coordinate analysis (PCoA) based on the unweighted (Figure 4A) and weighted (Figure 4B) UniFrac distance matrices. The gut microbiota was markedly altered as Py 17XL infection progressed in BALB/c mice. On the PCoA plot, each symbol represents the gut microbiota of one mouse in the relevant stage. The similarity degree between community structures verified by PCoA was examined by comparing within-group unweighted UniFrac distances for the PC1 axis among the Ctrl, PyD2, and PyD5 groups (Figure 4A). In contrast, the similarity degree between community structures verified by PCoA was examined by comparing within-group weighted UniFrac distances for the PC2 axis among the Ctrl, PyD2, and PyD5 groups (Figure 4B).

Following the PCoA plot, the between-group distances were significantly higher than the within-group distances for each group (ANOSIM, MRPP, *p* ≤ 0.01). The *R* value and *P* value of the ANOSIM between the Ctrl and PyD2 groups were 0.2386 and 0.002, respectively; the *R* value and *P* value of the ANOSIM between the Ctrl and PyD5 groups were 0.1739 and 0.004, respectively, and the *R* value and *P* value of the ANOSIM between PyD2 and PyD5 group were 0.3018 and 0.002, respectively. The *R* value indicated significant differences among the three groups, while the *P* value indicated statistical significance (*p* < 0.05). MRPP analysis (0.401, 0.384, 0.397; 0.422, 0.397, 0.423; observed-delta, expected-delta) indicated a lower difference within the groups and a higher difference between the groups. The *A* value of the MRPP was 0.05011, 0.03178, and 0.06291 (representing Ctrl-PyD2, Ctrl-PyD5, and PyD2-PyD5, respectively), demonstrating that the difference between groups was higher than that within groups (*A* > 0). Significance < 0.05 indicated a significant difference. These data demonstrated that the microbiota composition among Ctrl, PyD2 and PyD5 feces was significantly different.

#### Potential Biomarker Discovery

**FIGURE 5:**
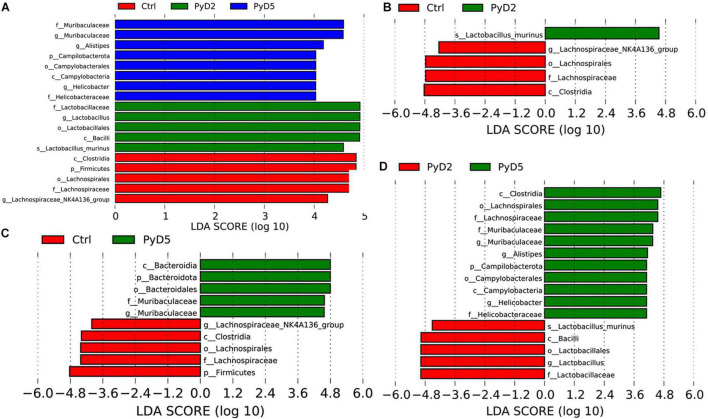
LEfSe identified the bacterial taxa significantly differentiated among different groups. Only taxa with the statistical differences between groups were shown in the figure, which was determined by meeting an LDA significant threshold > 4. **(A)** Histogram of the linear discriminant analysis (LDA) scores distribution among three groups; **(B)** Histogram of LDA score distribution between the Ctrl and PyD2 groups. **(C)** Histogram of LDA score distribution between the Ctrl and PyD5 groups. **(D)** Histogram of LDA score distribution between the PyD2 and PyD5 groups.

**FIGURE 6:**
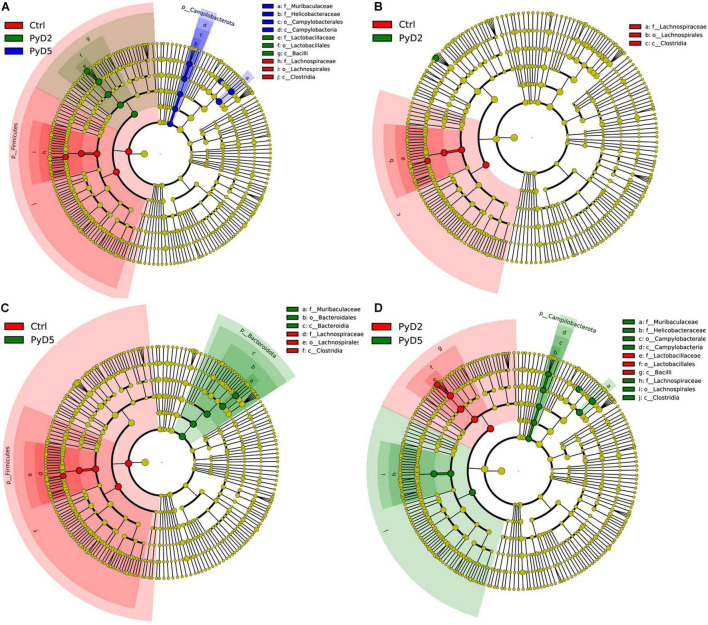
Cladogram of the most differentially abundant bacterial taxa among three groups. The red, green, and blue colors represent the bacterial communities of Ctrl, Py D2, and PyD5 groups, respectively. **(A)** Cladogram of the most differentially abundant bacterial taxa in the Ctrl, PyD2, and PyD5 groups. **(B)** Cladogram of the most differentially abundant bacterial taxa in the Ctrl and PyD2 groups. **(C)** Cladogram of the most differentially abundant bacterial taxa in the Ctrl and PyD5 groups. **(D)** Cladogram of the most differentially abundant bacterial taxa in the PyD2 and PyD5 groups.

LEfSe analysis of the top 10 taxa (Figure 5) identified the different statistical biomarkers, which were the microbial taxa with significant differences among the three groups. This threshold could obtain as many taxa as possible for significant comparisons and eliminate the rarest taxa in the analysis. The potential biomarkers at different taxonomic levels were determined in all three stages (Figures 6A-D). At the genus level, the biomarker with a significant difference between the PyD2 and Ctrl groups was *Lactobacillus* (*Lactobacillus murinus*, which belongs to the *Firmicutes* phylum and *Lactobacillaceae* family) (Figures 5A,B). The biomarker with a significant difference between the PyD5 and Ctrl groups was *Muribaculaceae* (belonging to the *Bacteroidetes* phylum and *Muribaculaceae* family) (Figures 5A,C). In contrast, the biomarkers with a considerable difference between PyD5 and PyD2 groups were *Muribaculaceae*, *Alistipes* (belonging to the *Bacteroidetes* phylum and *Rikenellaceae* family), and *Helicobacter* (belonging to the *Campilobacterota* phylum and *Helicobacteraceae* family) (Figures 5A,D).

Co-genetic network maps (Supplementary Figures 4, 5) were generated to identify the OTU co-abundance units and provide new insights for studying complex microbial community compositions and functions. These results presented an OTU co-abundance network approach to successfully generate associations that could generalize empirical data, and therefore, it might become a potential method for identifying microbiota associations. Co-genetic network maps can directly observe the effects on microbiota, the mutual advantage of the dominant species, and the related interactions of species after Py infection. Combined with the LEfSe of the top 10 taxa and networks, they showed that the genus *Lactobacillus* was associated with the genera *Muribaculaceae*, *Bacteroides*, *Alloprevotella*, and *Parabacteroides*. The genus *Alistipes* was correlated with the genera *Rikenella*, *Muribaculum*, *Odoribacter*, *Mucispirillum*, *Bacteroides*, and *Parabacteroides*. The genus *Muribaculaceae* was connected with the genera *Muribaculum*, NK4A214_group, *Clostridia*_UGG_010, and *Rikenellaceae*_RC9_gut_group. In contrast, the genus *Helicobacter* was associated with the genera *Mucispirillum*, *Lactobacillus*, and *Enterococcus*.

## Discussion

The rodent and avian *Plasmodium* parasites do not infect humans and are not a threat to public health (Su and Wu, 2021). However, they may have economic impacts (Grim et al., 2008). Therefore, it is meaningful to explore the interactional effect between rodent *Plasmodium* parasites and mice. This study demonstrates that *Plasmodium* rodent species infection can alter the murine gut microbiota composition. Specifically, the gut microbiota compositions in Py 17XL-infected BALB/c mice were altered compared with Py 17XL-uninfected mice. That is, malaria caused by *Plasmodium* might be associated with the alteration of the gut microbiota. Consistent with this thought, a study clarified a meaningful association between the host’s microbiota composition and the proleptic risk of *Plasmodium* infection (Yooseph et al., 2015; Waide et al., 2020).

Both species accumulation boxplots and rarefaction curves approached the saturation level, which demonstrated almost full coverage of the whole microbiota diversity. Based on alpha and beta diversity analysis, the diversity and richness of the intestinal microbiota in all fecal samples were altered after Py 17XL infection. In the present study, the gut microbiota at the phylum level was dominated by *Firmicutes* and *Bacteroidetes*, which were consistent with the previous survey (Villarino et al., 2016).

The current data also demonstrate that the relative richness of dominant taxa was altered after infection. The abundance of *Lactobacillaceae* and *Bifidobacteriaceae* was dramatically increased at 2 dpi and then slightly decreased at 5 dpi. A previous study manifested that among differences in the gut flora were increased abundances of *Lactobacillus* (member of the *Lactobacillaceae* family) and *Bifidobacterium* (member of the *Bifidobacteriaceae* family) in resistant mice (Villarino et al., 2016). At the genus level, the gut microbiota in the samples of infected mice was characterized by higher amounts of *Lactobacillus*, *Muribaculaceae*, *Bacteroides*, *Alistipes*, and *Helicobacter*. Meanwhile, LEfSe analysis was performed to obtain biomarkers with significant differences between Py 17XL-infected and -uninfected mice. The biomarker with a considerable difference between the PyD2 and Ctrl groups was *Lactobacillus* (*Lactobacillus murinus*). The genus *Lactobacillus* was indicated to play a role in the stress response in patients (Hemarajata and Versalovic, 2013; Aizawa et al., 2018). That is, the genus *Lactobacillus* in the gut microbiota is related to the disease. Hu et al. verified that IBD induced with DSS caused a decrease in *Lactobacillus* gut microbiota at the genus level (Hu et al., 2020). The biomarker with an obvious difference between the PyD5 and Ctrl groups was *Muribaculaceae*. Moreover, the biomarkers with a noticeable difference between the PyD5 and PyD2 groups were *Muribaculaceae*, *Alistipes*, and *Helicobacter*. Parker et al. (2020) indicated that the genus *Alistipes* in the gut microbiota might have protective effects against some diseases such as colitis, liver fibrosis, and cancer immunotherapy. Other studies have revealed *Alistipes* is pathogenic in colorectal cancer (Moschen et al., 2016), and it is associated with mental signs of depression (Parker et al., 2020). A previous study manifested that the species *Helicobacter pylori* played a potential protective role in IBD (Sjomina et al., 2017). Kubota-Aizawa et al. (2017) performed an epidemiological survey of gastric *Helicobacter* spp. that were related to gastrointestinal disease in dogs.

Meanwhile, co-genetic network maps were generated to identify the OTU co-abundance units. Combined with the LEfSe on top 10 taxa and networks, they indicated that the genus *Lactobacillus* was associated with the genera *Muribaculaceae*, *Bacteroides*, *Alloprevotella*, *Parabacteroides*, and so on. A previous study suggested the attractive probiotic potential of the genus *Bacteroides* (species *B*. *thetaiotaomicron*) in IBD (Sitkin and Pokrotnieks, 2019). Another study found that genes for *Bacteroides fragilis* toxin encoded secreted oncotoxins correlated with colorectal cancer (Dejea et al., 2018). Chen et al. (2020) discovered that *Bacteroides*, *Alistipes*, *Alloprevotella*, *Odoribacter*, and *Parabacteroides* were closely correlated with short-chain fatty acid (SCFA) production, such as propionate, butyrate, and acetate. Silva et al. (2018) considered that butyrate played an essential protective role in intestinal homeostasis and IBD. In contrast, Chakravarty et al. (2019) demonstrated that the intestinal SCFA composition did not explain the effects of gut microbiota on malaria severity. The genus *Alistipes* was correlated with the genera *Rikenella*, *Muribaculum*, *Odoribacter*, and *Mucispirillum*. The genus *Muribaculaceae* was associated with the genera *Muribaculum* and *Clostridia*_UGG_010. The genus *Helicobacter* was correlated with the genera *Mucispirillum* and *Lactobacillus*. A previous study demonstrated that *Rikenella* or Rikenellaceae had an inverse relationship with body weight or fat mass (Li et al., 2017; Zhou et al., 2018) in mice and humans. Also, a high abundance of *Rikenella* is associated with low chronic non-infective inflammation (Miranda-Ribera et al., 2019). Brugiroux et al. (2016) displayed that *Muribaculum intestinale* was a strictly anaerobic bacterium that inhibited the colonization of pathogenic bacteria and regulated the balance of bacteria. Gomez-Arango et al. (2016) verified that the genus *Odoribacter* was known as a butyrate producer. A previous study reported that *Mucispirillum schaedleri* was abundant in the intestinal mucus layer of mice and humans, and that it played an essential role in inflammation (Loy et al., 2017). Besides, Lin et al. (2019) considered that patients with Parkinson’s disease had an increased abundance of *M. schaedleri* in the fecal microbiota. Schönherr-Hellec and Aires (2019) demonstrated that *Clostridia* species were responsible for necrotizing enterocolitis.

A previous study indicated that using probiotics might be a potential method to limit the reproduction of pathogenic microbiota (Pickard et al., 2017). These findings offered more insights into host gut microbiota alterations after Py 17XL infection and showed that microbiota analysis would play an important role in the early diagnosis of fatal malaria and in perceiving the pathogenesis of malaria. LEfSe analysis combined with the co-genetic OTU network showed that the genus *Alistipes* was a potential biomarker that may play a meaningful role in the pathogenesis and progression of Py malaria. The anaerobic bacterium *Alistipes* is found almost in the healthy human gastrointestinal tract (Shkoporov et al., 2015), and it is pathogenic in colorectal cancer (Moschen et al., 2016) and is associated with depression (Bangsgaard Bendtsen et al., 2012). These data indicated gut microbiota modulation can regard as a novel method of preventing severe malaria, which is consistent with the insight of Waide (Waide and Schmidt, 2020).

This article studied the alteration of the gut microbiota profile in BALB/c mice induced by Py 17XL infection and obtained potential biomarkers correlated with murine malaria. However, this research also had several limitations. In future studies, it will be important to explore the role of microbiota in the progress of malaria and to evaluate the microbiota modulation interfere with protection and less severity of the disease in a non-lethal infection model as PbA or Py 17XNL in BALB/c mice, and to assess the role of the microbiota more robustly using experimental approaches, such as non-lethal infection models, antimalarial drug therapy, and GF fecal microbiota transplantation.

The gut microbiota in mice before and after Py 17XL infection was characterized, and the overrepresented microbial taxa in infected and uninfected mice were identified. Meanwhile, several potential biomarkers at different taxonomic levels were obtained from this study. These microbiota taxa may serve as direct targets to clarify their roles in the pathogenesis and progression of lethal malaria in future studies. Also, in an association study of Py 17XL infection risk and gut microbiota composition, gut microbiota composition modulation will decrease Py 17XL infection risk and may be regarded as a standard to some antimalarial drugs or malaria vaccines.

## Data Availability Statement

The datasets presented in this study can be found in online repositories. The names of the repository/repositories and accession number(s) can be found below website: https://www.ncbi.nlm.nih.gov/sra/PRJNA798515.

## Ethics Statement

The animal study was reviewed and approved by the Institutional Animal Care and Use Committee of the Hubei University of Medicine under permit number HBMU-S20160414.

## Author Contributions

WG and JL conceived the study, participated in its design, performed the data analysis and interpretation, and drafted the manuscript. WG, SY, and JL carried out the experiments. XS, HZ, and FL participated in analyzing and interpreting the data. All authors contributed to the article and approved the submitted version.

## Conflict of Interest

The authors declare that the research was conducted in the absence of any commercial or financial relationships that could be construed as a potential conflict of interest.

## Publisher’s Note

All claims expressed in this article are solely those of the authors and do not necessarily represent those of their affiliated organizations, or those of the publisher, the editors and the reviewers. Any product that may be evaluated in this article, or claim that may be made by its manufacturer, is not guaranteed or endorsed by the publisher.
