# Supplementary figures and images for: Observation of the Gut Microbiota Profile in BALB/c Mice Induced by Plasmodium yoelii 17XL Infection
Source: Front Microbiol. 2022 Mar 31;13:858897. doi: 10.3389/fmicb.2022.858897 (PMC9009211; doi:10.3389/fmicb.2022.858897)

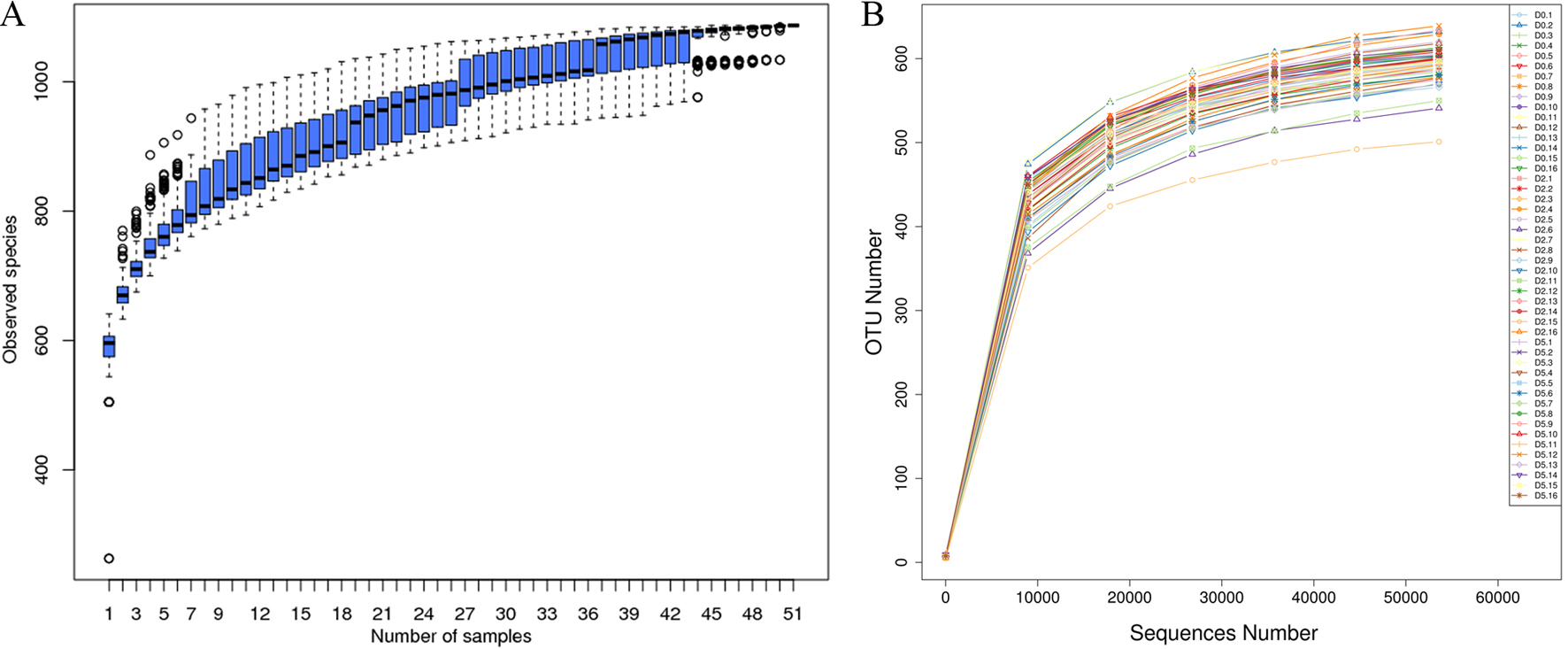

Supplement: Supplementary file 2 [file Image_1.JPEG]

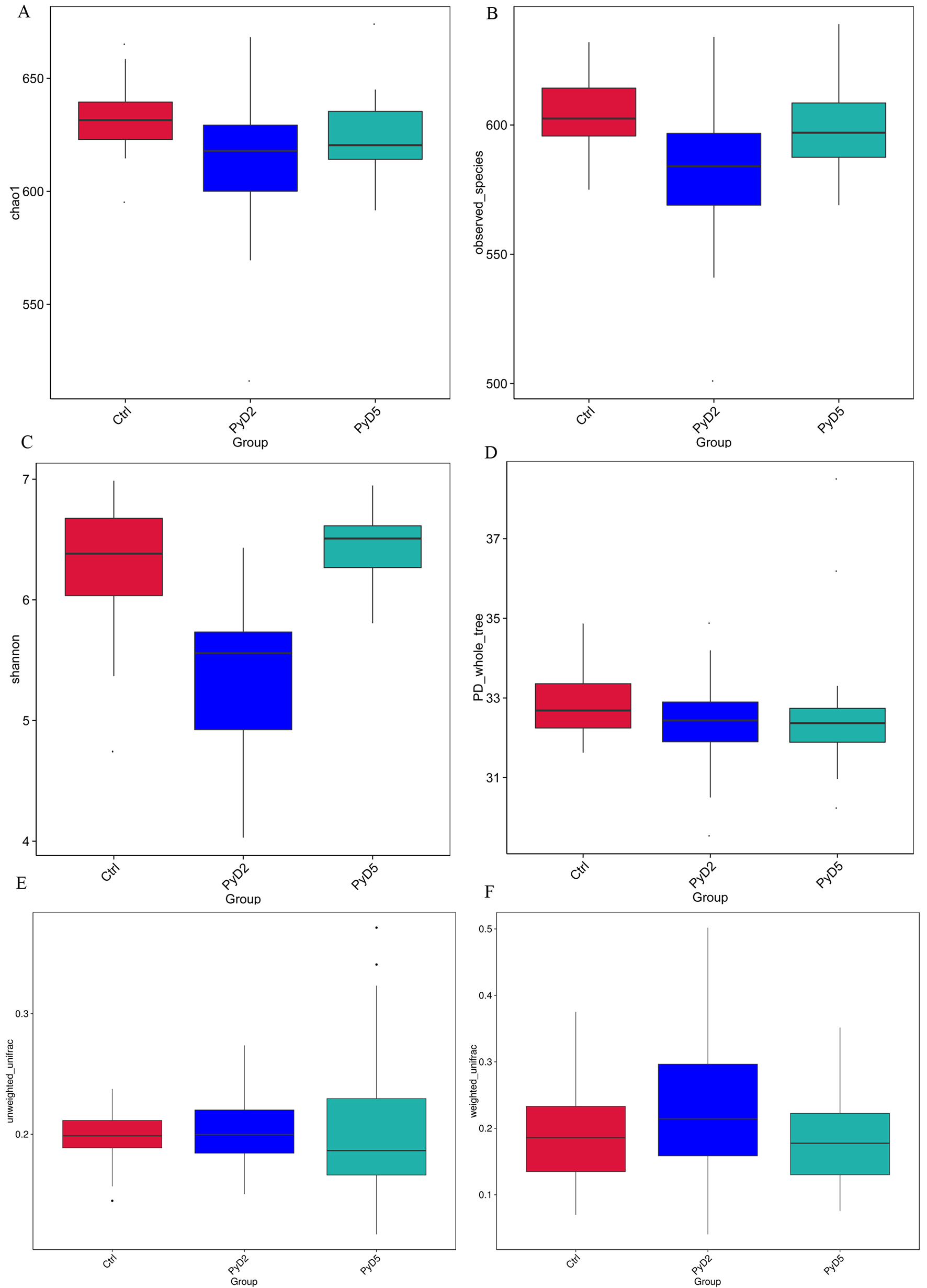

Supplement: Supplementary file 3 [file Image_2.JPEG]

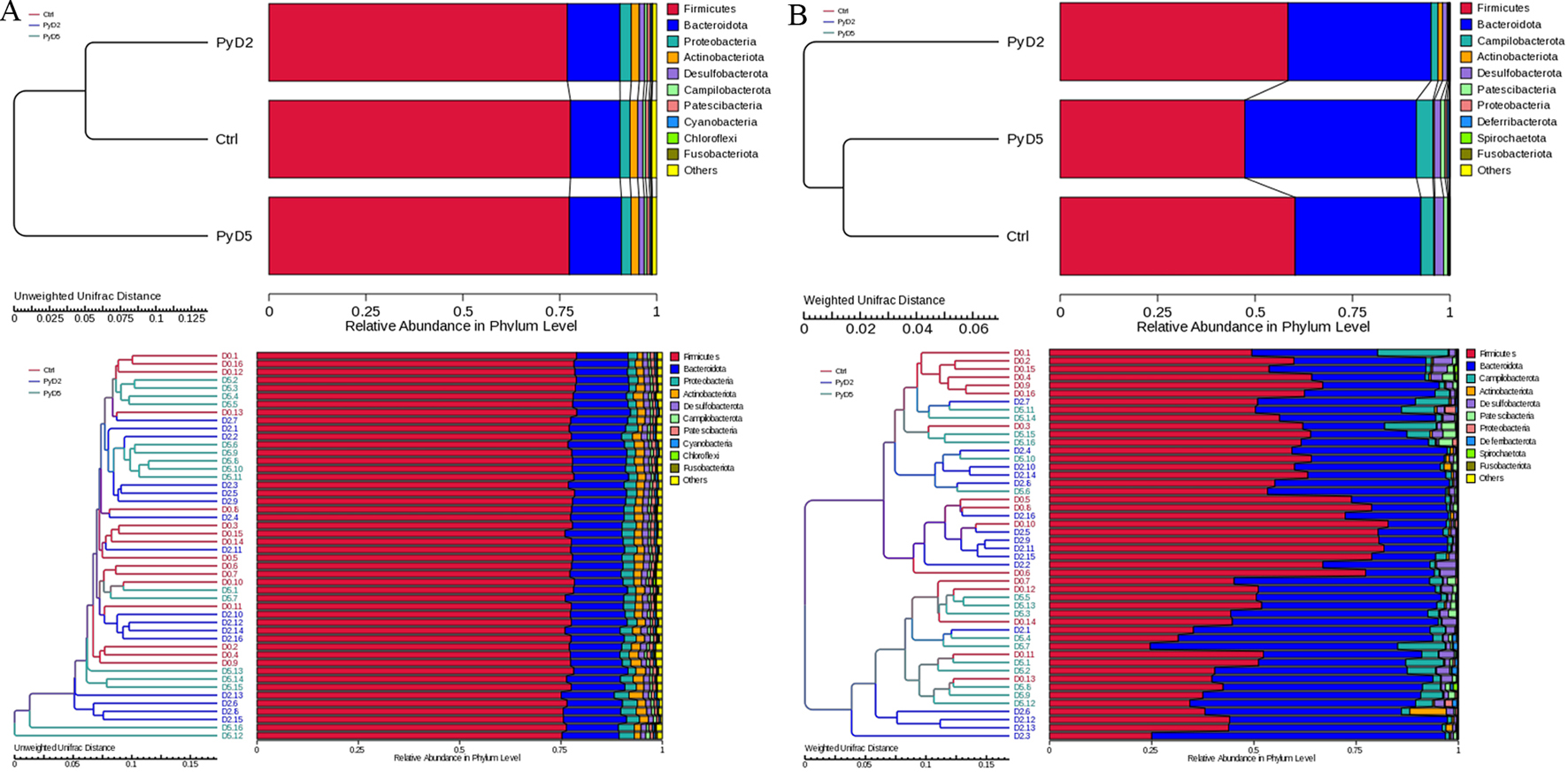

Supplement: Supplementary file 4 [file Image_3.JPEG]

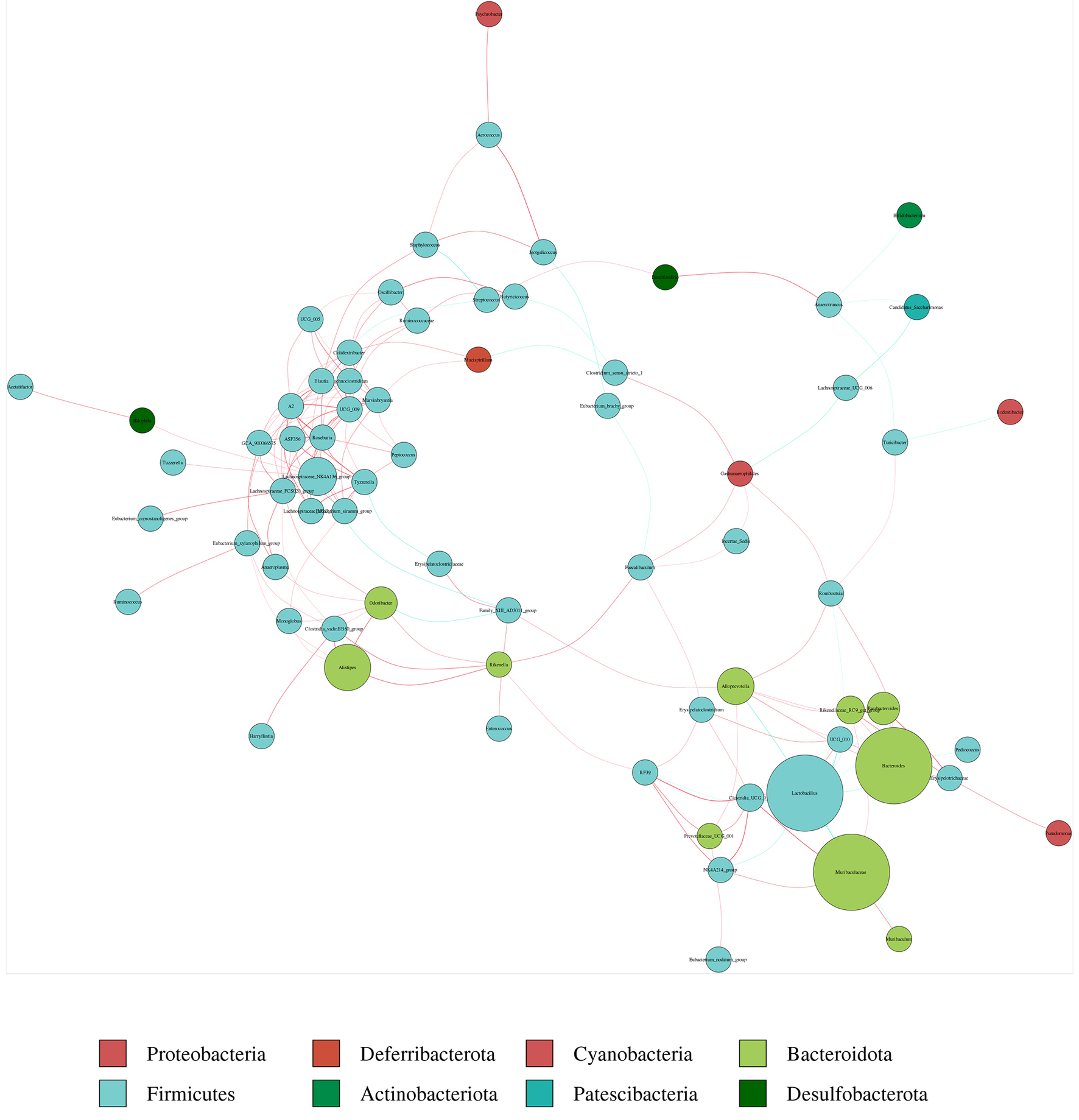

Supplement: Supplementary file 5 [file Image_4.JPEG]

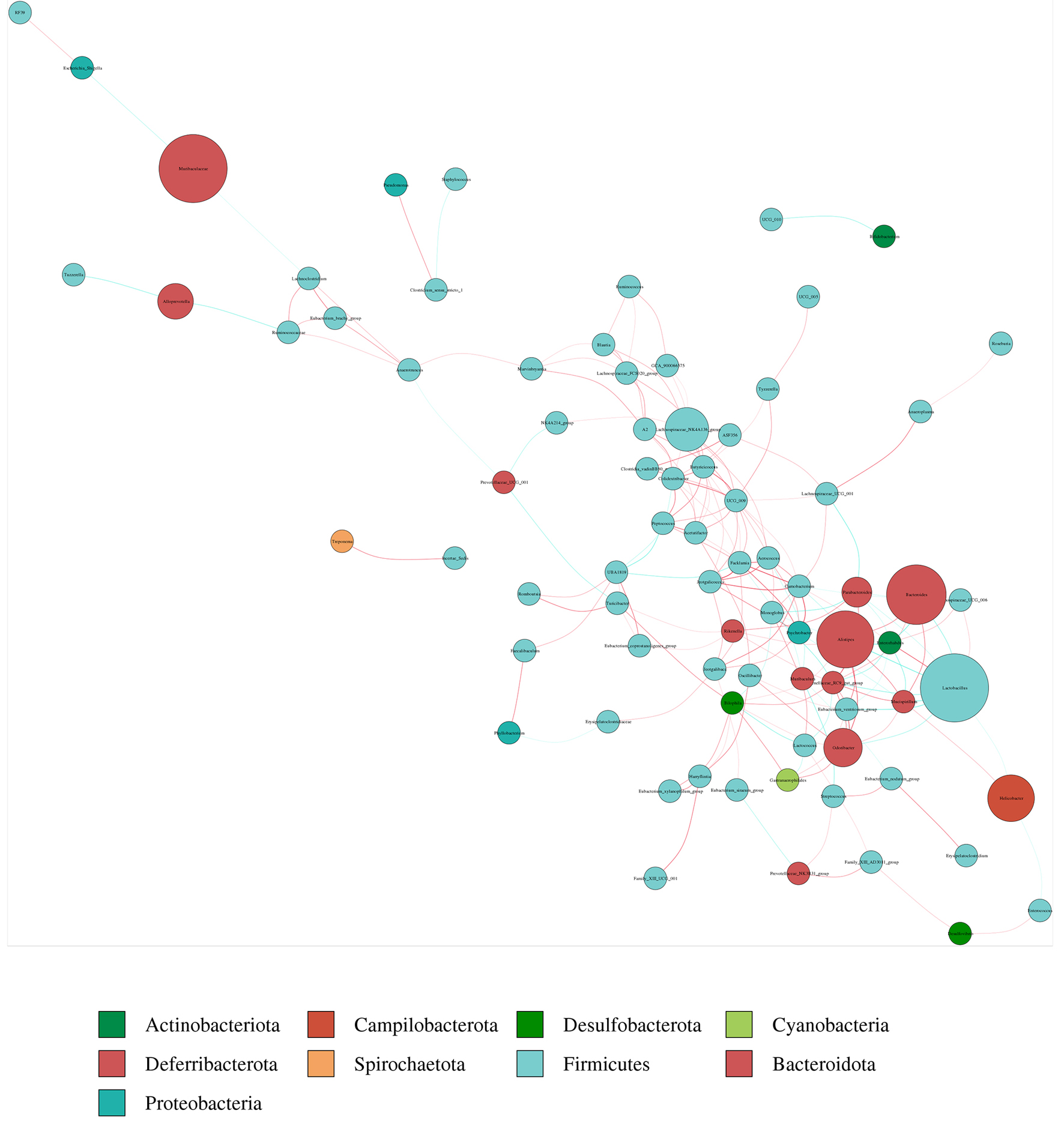

Supplement: Supplementary file 6 [file Image_5.JPEG]
